# Enhanced Extraction Technique of Omarigliptin from Human Plasma—Applied to Biological Samples from Healthy Human Volunteers

**DOI:** 10.3390/molecules25184232

**Published:** 2020-09-15

**Authors:** Shereen Mowaka, Nermeen Ashoush, Mariam Tadros, Noha El Zahar, Bassam Ayoub

**Affiliations:** 1 Pharmaceutical Chemistry Department, Faculty of Pharmacy, The British University in Egypt, El-Sherouk City, Cairo 11837, Egypt; shereen.hassib@bue.edu.eg; 2 The Center for Drug Research and Development (CDRD), Faculty of Pharmacy, The British University in Egypt, El-Sherouk City, Cairo 11837, Egypt; nermeen.ashoush@bue.edu.eg; 3 Analytical Chemistry Department, Faculty of Pharmacy, Helwan University, Ain Helwan, Cairo 11795, Egypt; 4 Clinical Pharmacy and Pharmacy Practice Department, Faculty of Pharmacy, The British University in Egypt, El-Sherouk City, Cairo 11837, Egypt; 5 Pharmaceutical Analytical Chemistry Department, Faculty of Pharmacy, Ain Shams University, Organization of African Unity Street, Abassia, Cairo 11566, Egypt; mariam.tadros@pharma.asu.edu.eg (M.T.); nohamorsyelzahar@pharma.asu.edu.eg (N.E.Z.)

**Keywords:** enhanced extraction, human plasma, human volunteers, LC-MS/MS, omarigliptin

## Abstract

Enhancing drug extraction from human plasma is a challenging approach that critically affects pharmacokinetic and any further clinical studies based on the drug C_min_ and C_max_ values. It also has a serious impact on the sensitivity and the lower limit of quantification (LLOQ) value of the bio-analytical methods. An advanced liquid chromatography tandem mass spectrometry (LC-MS/MS) bio-analytical method of omarigliptin (25–1000 nM) was established in human plasma using one-step liquid-liquid extraction. Alogliptin was used as an internal standard (IS) to attain good recovery and reproducibility while reducing the effects of the matrix. Enhanced plasma extraction of omarigliptin was successfully achieved with tertiary butyl methyl ether—diethyl ether (TBME-DEE) mixture as the extracting solvent, while using acetonitrile as the diluent solvent for the IS to effectively decrease the formed emulsion. Multiple Reaction Monitoring (MRM) of the transition pairs of *m*/*z* 399.2 to 153.0 for omarigliptin and *m*/*z* 340.2 to 116.0 for alogliptin was employed in positive Electro Spray Ionization (ESI) mode. Human plasma samples were collected after 1.5 h (t_max_) of Marizev^®^ (12.5 mg) tablets administration to healthy human volunteers showing average concentration of 292.18 nM. Validation results were all satisfactory including successful stability studies with bias below 12%. The proposed study will be valuable for ethnicity comparison studies that will be commenced on omarigliptin in Egypt by the authors in prospective study, following the FDA recommends, to evaluate possible sub-group dissimilarities that include pharmacokinetic parameters.

## 1. Introduction

Diabetes Mellitus prevalence increases constantly worldwide. Moreover, it is worthy to mention that many recent COVID-19 studies reported that Diabetes Mellitus was a major contributing factor either for non-survivals and/or hospitalization. Diabetic patients represented 12% of non-survivors in a study and represented 22% of the hospitalized patients in another study [1,2]. Generally, people with Diabetes Mellitus are most likely to suffer from different complications when infected with virus ranging from mild to severe. Unmanaged levels of blood glucose elevate the risk and the severity of any common respiratory attack [3,4]. Uncontrolled Diabetes Mellitus leads to a weak immunity response, as the body becomes no more able to fight the infection [1,2]. Hence, it becomes imperative to find better therapeutic strategies for glycemic control.

**Figure 1:**
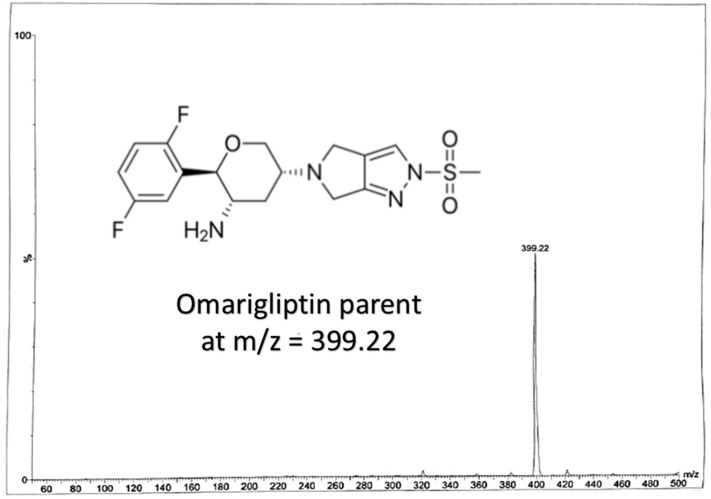
Chemical structure of omarigliptin and its full scan mass spectrum.

Gliptins are considered to be effective agents for the treatment of type 2 Diabetes Mellitus. Omariglitpin (OTN), Figure 1, is a long-acting once weekly administered antidiabetic drug acting as dipeptidyl peptidase-4 (DPP-4) inhibitor [5,6,7]. It has been licensed for use in Japan since 2015 but its phase III development in US has been halted for undisclosed commercial reasons [5]. It improved the glycated hemoglobin A1c (HbA1c) as it reversibly inhibits DPP-4 enzyme, which prolongs the circulating half-life of glucagon-like peptide-1 (GLP-1) increasing insulin secretion. Contrary to the once-daily administered DPP-4 inhibitors available in market, once-weekly administered OTN can improve patients’ compliance to the treatment protocol [5,6,7,8,9].

Literature review reveals that OTN pharmacokinetics parameters were studied with renal impairment [10], in repositioning studies [11] and in healthy Japanese volunteers [12]. OTN pharmacokinetics were investigating the supra-therapeutic dose of OTN [13] and the effect of age, sex and obesity [14]. Clinical studies were also concerned with the effects of multiple and chronic dosage regimens [15,16]. Although, these many articles deal with the pharmacokinetics and/or bio-analysis of OTN [10,11,12,13,14,15,16], most of the reported studies did not discuss the full details of the validated bio-analytical procedure that may be attributed to using the same methods for many clinical studies by the same common authors [12,13,14,15,16,17]. They were emphasizing the findings of the main pharmacokinetic parameters rather than developing or validating a bio-analytical procedure. One liquid chromatography tandem mass spectrometry (LC-MS/MS) method was discussed in full details for OTN bio-analysis [18] but that method was related to rats’ plasma only and the authors used only direct precipitation with acetonitrile for the extraction of OTN from spiked and biological rats’ plasma samples. The authors of the current study modified the previously mentioned rats’ plasma method [18] in another repositioning publication [11] but with direct precipitation with acetonitrile. The lower limit of quantification (LLOQ) was 50 ng/mL which is very high in comparison to the LLOQ in the current work with the enhanced extraction technique. Moreover, the same authors reported in another UPLC study that the UV detector failed to detect OTN in human plasma due to interference [19].

Enhancing the extraction techniques for new drugs enrich the literature and opens the door for further studies. Hence, the purpose of our study is to focus on the bio-analysis of OTN using LC-MS/MS by means of an enhanced one-step liquid-liquid extraction of OTN from human plasma followed by vacuum evaporation and then reconstitution. The bioanalytical method was validated as per FDA guidelines to include linearity, selectivity, accuracy, precision, stability and matrix factor [20].

## 2. Methods

### 2.1. Chemicals and Reagents

Six different batches of blank human plasma, OTN raw material (99.0%), Alogliptin raw material, internal standard (IS) (99.2%), Marizev^®^ (12.5 mg) tablets, tertiary butyl methyl ether (TBME), diethyl ether (DEE) were kindly donated by the British University in Egypt (CDRD, BUE) based on previous research collaborations (Repositioning CDRD-BUE project). Acetonitrile, methanol, and water (all of HPLC grade) and formic acid were obtained from (Sigma, St. Louis, MO, USA).

### 2.2. LC-MS/MS Conditions

Some of the coming conditions were adopted from the authors’ previous LC-MS/MS work (published repositioning study on OTN and IS using direct precipitation for rats’ plasma [11]). LC-MS/MS was performed via Waters^®^ UPLC-TQ with Electro Spray Ionization (ESI) (USA), Mass Lynx software (4.1 version) and Agilent C_18_ column (1.8 µm, 50 × 2.1 mm). An isocratic mobile phase of acetonitrile/0.3% formic acid (90:10, *v*/*v*), filtered via a 0.2 μm filter membrane degassed for 25 min, ten microliters as the injection volume, 0.3 mL/min as the selected flow rate to decrease the retention time and provide a faster method and 1.2 min as the run time for OTN bioassay, were applied. The column temperature was set at 25 °C. The mass spectrometer parameters included the adjustment of cone voltage values to 40 V and 30 V and collision energy values to 50 eV and 55 eV for OTN and IS, respectively. Multiple Reaction Monitoring (MRM) of *m*/*z* 399.2 to 153.0 for OTN and *m*/*z* 340.2 to 116.0 for IS in the ESI positive mode was implemented. Further MS parameters were adjusted including turbo ions spray at 400 °C, capillary temperature at 275 °C, sheath and auxiliary gas at 15 and 2 psi, respectively, ion spray voltage of 3800 V, capillary voltage of 4 KV, capillary offset of 35 and desolvating line temperature at 400 °C.

### 2.3. Calibrators, QC Samples and Sample Preparation

OTN standard stock solution in methanol was prepared as (1 mM) then working solutions with different concentrations were prepared in methanol (2.5, 5, 10, 40, 50, 70, 80 and 100 µM). 10 μL of each one of the prepared working solutions was used to spike 990 μL blank plasma to prepare calibrators and QC samples as 25 nM (LLOQ), 50, 100, 400, 500 nM (Medium quality control sample, MQC) and 700, 800 (High quality control sample, HQC) and 1000 nM. An aliquot of 250 μL of each plasma sample was spiked with 100 μL of the IS in acetonitrile (300 nM), liquid-liquid extracted by 1.5 mL of a mixture (50:50, *v/v*) of TBME and DEE for 15 min centrifugation at 15,000 rpm and then frozen at −80 °C. 1.3 mL of the upper organic level was vacuum evaporated till dryness, reconstituted with 250 μL methanol and injected into the auto-sampler. Peak area ratios to IS was used against concentrations to predict the calibration curve and the regression equation.

### 2.4. Bioanalytical Validation

FDA guidelines [20] were followed for 6 calibrators to predict the linearity while LLOQ, MQC, HQC levels (n = 5) were used to evaluate both accuracy and precision five times a day and on three successive days. The bias value, standard deviation (S.D.) and % RSD were calculated. Six different batches of blank plasma (from different sources) donated form (CDRD-BUE) were checked for interference as a measure of selectivity. Injection of high values of concentration directly after the blank samples was used to ensure the absence of a significant carry over. Matrix factor was estimated by calculating the ratio of the peak area in the presence of the matrix components of human plasma (blank matrix as human plasma sample spiked after extraction with the drug under investigation, OTN), to the peak area in absence of the human plasma matrix components (solution of the drug, OTN). Comparing the area ratios under the peak of the post extracted samples to the neat standards was used to calculate the matrix factor while comparing the area ratios under the peak of the underlying extracted samples to the post extracted samples was used to calculate the extraction recovery. Stability of LLOQ and HQC was estimated based on 4 different bio-assays after leaving the samples 3 h in the auto-sampler or leaving them 3 h at room temperature (bench top stability) or analyzing them after three complete cycles of both freeze and thaw. Finally, stability for the long term was investigated by checking the samples after two weeks while freezing at −80 °C.

### 2.5. Biological Samples and Ethical Approval

After the approval of the British University in Egypt ethical committee (Code: CL/2003, March 2020) and after submission of the proposed LC-MS/MS study to clinicaltrials.gov (ID: NCT04365907), one mL blood samples from 4 volunteers were collected after 1.5 h of the oral administration of the drug (Marizev^®^ as 12.5 mg of OTN). Then, centrifugation at 3000 rpm was performed to extract the plasma. This experimental trial was performed in order to employ the developed method for the determination of OTN in human plasma.

## 3. Results and Discussion

The proposed study will be valuable for ethnicity comparison studies that will be commenced on OTN in Egypt by the authors in a prospective study. This study will cover one week bio-analytical assays after its administration as the FDA recommends [20] and will compare results across pharmacokinetic studies to evaluate possible sub-group dissimilarities. Ethnic difference has been taken in consideration in recent years. Typically, global companies start clinical development in Japan after the United States and Europe, some genetic factors could explain a significant proportion of dose variability of many drugs between different ethnic groups [21,22]. The recognition of racial differences in disease outcomes and many investigations have identified genetic factors that explain a significant proportion of dose variability. This evidence underpins the prevailing hypothesis that genotype guided therapy should improve dosing accuracy [21,22] that requires validated bioanalytical studies to be used for the comparative pharmacokinetic studies. Therefore, an advanced analytical technique and enhanced extraction of drugs from human plasma become a challenging approach that greatly affects pharmacokinetics, other clinical studies based on the drug C_min_ and C_max_ values and the bio-analytical methods sensitivity.

**Figure 2:**
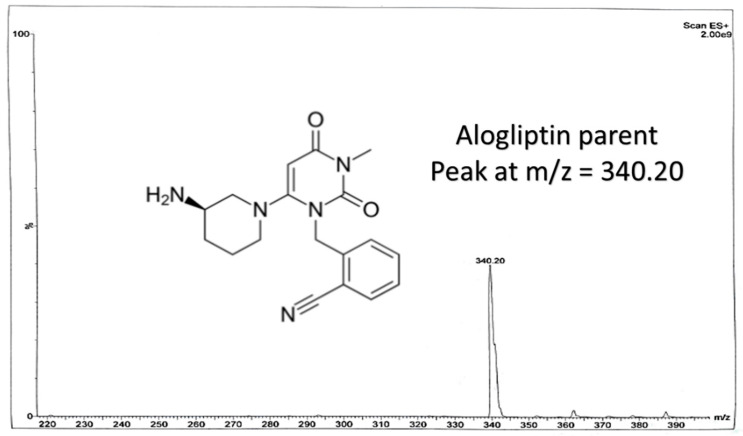
Chemical structure of alogliptin (IS) and its full scan mass spectrum.

**Figure 3:**
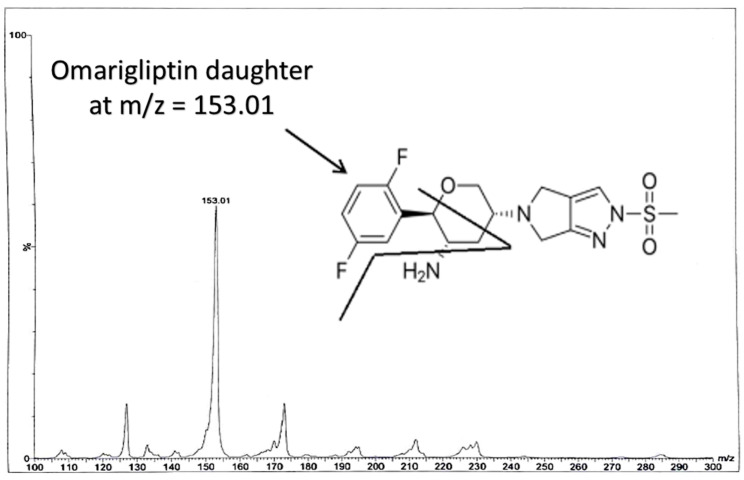
Daughter ions mass spectra in positive Electro Spray Ionization (ESI) ion detection mode with the proposed fragments showing *m*/*z* at 153.01 for omarigliptin.

**Figure 4:**
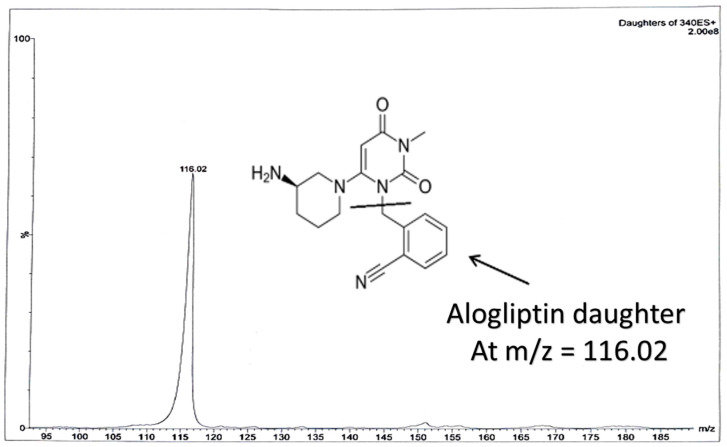
Daughter ions mass spectra in positive ESI ion detection mode with the proposed fragments showing *m*/*z* at 116.02 for alogliptin.

In our study, effective liquid-liquid extraction based on the use of TBME-DEE mixture as the extracting solvent and vacuum evaporation followed by reconstitution, was established for the extraction of OTN from human plasma. Enhanced extraction was successfully achieved after using acetonitrile as the diluent solvent for the IS decreasing the emulsion formed due to the addition of the aqueous immiscible organic solvent TBME [23]. Therefore, using an appropriate mixture of the extracting solvent accompanied with high volume of acetonitrile; decreased the formed emulsion especially after freezing, which enabled the accurate withdrawing of up to 1.3 mL from the upper clear organic layer without interference from the emulsion intermediate layer. Adding acetonitrile (that contained the IS) was crucial as it decreased the usual resulting emulsion from mixing the immiscible extracting organic solvent and the plasma sample. Changing the ratio of the TBME-DEE mixture to (60–40%) or (40–60%), respectively did not affect the readings in the preliminary trials. LC-MS/MS bio-assay of OTN (25–1000 nM) was achieved in human plasma using alogliptin as IS. MRM function of the transition pairs of *m*/*z* 399.2 to 153.0 for OTN and *m*/*z* 340.2 to 116.0 for alogliptin was employed utilizing positive ESI mode, as shown in Figure 1, Figure 2, Figure 3 and Figure 4. The transition pairs were also used to verify the identity of the analyte whereas no other qualifying ion transitions were detected or found significant to be used. The use of glass tubes was avoided throughout the investigation especially for vacuum evaporation, centrifugation and reconstitution to avoid the reported gliptins’ adhesion [24] based on previous work by the authors on different gliptins in lab. The authors mentioned in the current study modified the rats’ plasma method [18] in another repositioning publication [11] but with direct precipitation with acetonitrile, the LLOQ was 50 ng/mL which is very high in comparison to the LLOQ in the current work with the enhanced extraction technique (9.98 ng/mL equivalent to 25 nM). The LLOQ in the current investigation is less than five times of the previous LLOQ [11]. Moreover, the same authors in another UPLC study [19], showed that the UV detector failed to detect OTN in human plasma due to interference from plasma endogenous components with the UV detector. They recommended LC-MS/MS accompanied with liquid-liquid extraction/vacuum evaporation to enhance the extraction procedure which is the case in the current study. A comparison between all the previously reported methods for OTN extraction and determination in plasma either rats’ or human plasma, as shown in Table 1, was implemented. In spite of some common authors reported lower LLOQ (2 ng/mL) in human plasma using ethyl acetate (after pH adjustment) in liquid-liquid extraction [12,13,14,15,16,17], they did not mention the full validation study for the bioanalytical procedure. No data regarding stability studies, selectivity, carry over, extraction recovery, method development, full detailed chromatographic procedure and/or matrix effect was reported. Furthermore, the authors of the current investigation did not prefer to use the pH adjustment step that was reported in the previous LC-MS/MS method [12,13,14,15,16,17] as it did not show significant difference after using TBME-DEE mixture with the satisfying full validation bioanalytical results.

**Figure 5:**
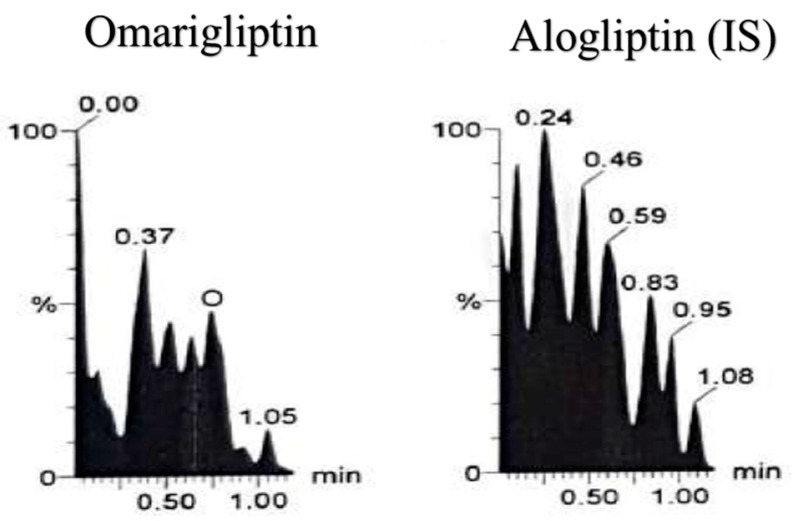
Blank plasma sample using liquid chromatography tandem mass spectrometry (LC-MS/MS).

**Figure 6:**
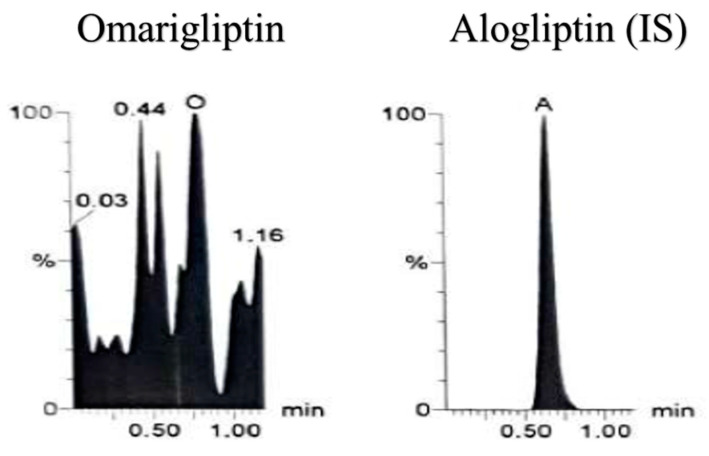
Zero plasma sample (spiked with IS) using LC-MS/MS showing only the IS using Multiple reaction monitoring (MRM) chromatogram of omarigliptin (*m*/*z* = 399.2 to 153.0) and alogliptin (IS, *m*/*z* = 340.2 to 116.0).

**Figure 7:**
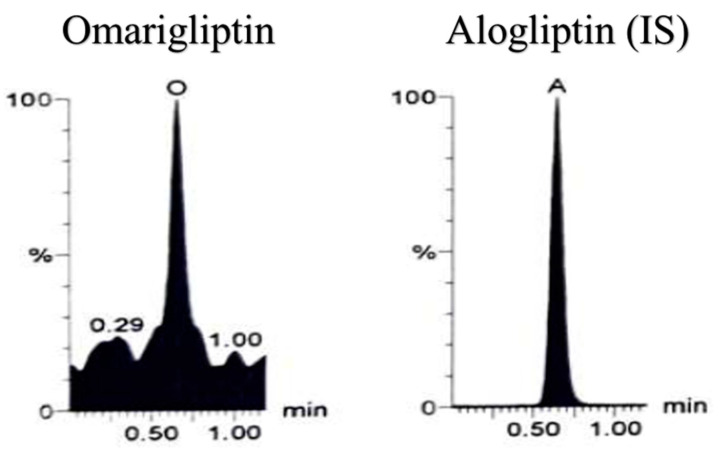
Lower limit of quantification (LLOQ) plasma sample using LC-MS/MS (25 nM of Omarigliptin) using Multiple reaction monitoring (MRM) chromatogram of omarigliptin (*m*/*z* = 399.2 to 153.0) and alogliptin (IS, *m*/*z* = 340.2 to 116.0).

Calibration (25–1000 nM) and full detailed validation outcomes were satisfactory with FDA bio-analytical guidelines [20]. Adequate method selectivity from six different batches of blank plasma was designated where no significant interference was observed among the MRM channels in blank (Figure 5), zero samples with IS (Figure 6) and reasonable outcomes at the LLOQ level of 25 nM (Figure 7) and no significant carry over was detected. The authors used LC-MS/MS technique as a detection technique rather than the separation strategy and based on their experience with chromatography, they ensured, while developing the LC method, that both the drug and the IS did not elute on the dead volume time before applying the sequence order in the instrument. The equation of the calibration curve was; y = 0.0033x − 0.0701, r = 0.9998 showing the good linearity of the applied method. Accuracy (n = 5) and precision (n = 15) were within ±20% as shown in (Table 2). Extraction recovery was 87.5% for the LLOQ and 89.66% for the HQC sample. Matrix factor denoting the effect of the matrix on the signal response and the ionization efficiency through matrix enhancement and/or suppression was evaluated. It was ranged from 81.4% to 86.24% indicating ion suppression for all concentrations of OTN in plasma. Stability measurements mentioned under methods showed recoveries more than 85% from the time-zero control which indicates OTN stability can be maintained through the sample treatment and storage.

**Figure 8:**
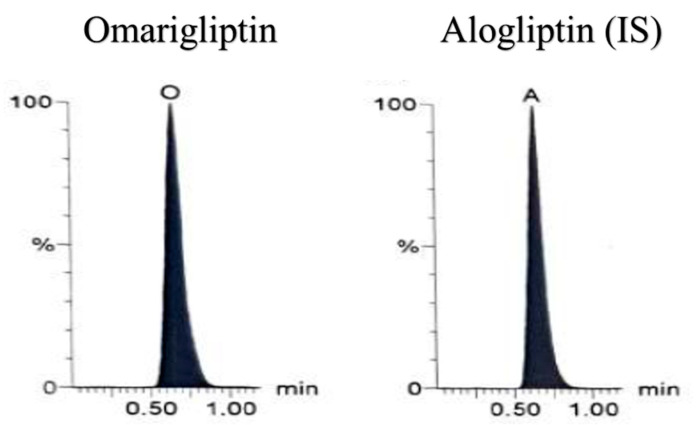
Multiple reaction monitoring (MRM) chromatogram of omarigliptin (*m*/*z* = 399.2 to 153.0) and alogliptin (IS, *m*/*z* = 340.2 to 116.0) in human plasma sample obtained 1.5 h after oral administration of one Marizev^®^ tablet (12.5 mg).

Eventually, OTN validated bioanalytical method was efficaciously applied to analyze human plasma samples. They were collected after 1.5 h (t_max_) of Marizev^®^ (12.5 mg) tablets administration to healthy human volunteers (as shown in Figure 8) showing average concentration of 292.18 nM, standard deviation of 21.1 nM and Percent relative standard deviation of 7.22%.

## 4. Conclusions

Working on anti-diabetic drugs’ bio-analysis is of great importance in the current days. It opens the door for ethnicity comparisons related to more pharmacokinetic bioequivalence studies and drugs’ approval in more countries leading to better HbA1c control and other outcomes. Bio-analysis of OTN (25–1000 nM) using LC-MS/MS was established as per FDA guidelines. Liquid-liquid extraction based on TBME-DEE mixture without the need of pH adjustment and vacuum evaporation followed by reconstitution, was implemented. Then, enhanced extraction of OTN from human plasma was achieved successfully after using acetonitrile as the diluent solvent for the IS to decrease the formed emulsion. The effective liquid-liquid extraction showed more sensitive results than direct precipitation. The LLOQ in the current investigation is less than five times of the previous LLOQ reported by the same authors. Enhancing the extraction techniques for new drugs enrich the literature and opens the door for further pharmacokinetic and bioequivalence studies.

## Figures and Tables

**Table 1:** Comparison between previously reported methods for omariglitpin (OTN) extraction and determination in plasma either rats’ plasma or human plasma.

Method	Extraction	LLOQ	Application	Reference
LC-MS/MS	Liquid-Liquid using TBME-DEE	25 nM (9.96 ng/mL)	Human plasma	Underlying investigation
LC-MS/MS	Direct precipitation using Acetonitrile	50 ng/mL	Rats’ plasma	[11]
LC-MS/MS	Liquid-Liquid using Ethyl acetate after pH adjustment	2 ng/mL	Human plasma	[12,13,14,15,16,17]
LC-MS/MS	Direct precipitation using Acetonitrile	4 ng/mL	Rats’ plasma	[18]
UPLC-UV	Liquid-Liquid using DEE	2.5 µg/mL	Rats’ plasma	[19]

**Table 2:** Accuracy and precision results for OTN determination by the proposed LC-MS/MS method.

Accuracy and Precision (n = 5, Three days)		LLOQ (25 nM)	MQC (500 nM)	HQC (800 nM)
**1st day Intraday**	Bias (mean, n = 5)	15.41	−6.30	−1.09
	Average Percent Recovery	115.41%	93.70%	98.91%
	S.D.	14.55	2.01	3.57
	% R.S.D	12.61	2.14	3.64
**2nd day Intraday**	Bias (mean, n = 5)	10.34	1.49	−0.10
	Average Percent Recovery	110.34%	101.49%	99.90%
	S.D.	15.37	1.90	1.82
	% R.S.D	13.92	1.87	1.82
**3rd day Intraday**	Bias (mean, n = 5)	13.80	0.98	−0.91
	Average Percent Recovery	113.80%	100.98%	99.09%
	S.D.	9.57	1.83	2.62
	% R.S.D	8.41	1.81	2.64
**Interday**	Bias (mean, n = 15)	13.40	−1.30	−1.01
	Average Percent Recovery	113.40%	98.70%	98.99%
	S.D.	13.41	4.02	2.89
	% R.S.D	11.83	4.07	2.92
